# Making space for street sport: an exploration of public value creation through the GAME House concept in Danish municipalities

**DOI:** 10.3389/fspor.2026.1867534

**Published:** 2026-10-14

**Authors:** Signe Højbjerre Larsen, Evald Bundgaard Iversen

**Affiliations:** Department of Sports Science and Clinical Biomechanics, University of Southern Denmark, Odense, Denmark

**Keywords:** facility management, informal sport, lifestyle sport, public value, social inclusion, sport facilities, sport policy, street sports

## Abstract

Street sports have gained increasing policy attention, challenging traditional sport infrastructures and governance models. This article examines how the Danish GAME House concept—publicly funded, hybrid indoor–outdoor facilities for street sport and urban culture—creates public value for communities often marginalized within conventional sports provision. The study draws on a qualitative case study of three GAME Houses in Denmark, combining document analysis with interviews with user groups, facility managers, and municipal actors. The analysis is guided by Mark Moore's theory of public value, using the strategic triangle as an analytical framework. The findings show that the GAME Houses generate public value by providing purpose-built facilities that meet the practical and cultural needs of street sport communities, strengthen local activity-based communities, and publicly recognize street sports as legitimate forms of participation. However, tensions emerge around operational capacity, user involvement, and the institutionalization of informal practices. The article concludes that public value creation in street sport facilities is context-dependent and relies on flexible governance, local adaptation, and ongoing negotiation between institutional support and community autonomy.

## Introduction

Street sports have moved from the margins to the mainstream, challenging sport policy and traditional sport infrastructure. Facilities for street sports and other kinds of lifestyle sports have become increasingly widespread around the world. These facilities, such as skateparks, parkour, and multi-purpose ball courts, are often located in urban outdoor environments, but increasingly also indoors—a trend described as the “indoorisation” of outdoor sports (1). The rise of street sports facilities has been described as driven by several factors, including general changes in young people's sports participation and everyday life, the need to create informal, low-barrier opportunities for participation (2) and the growing recognition of the potential of street sport to foster social inclusion and community engagement (3). In Denmark, a national survey from 2020 indicates that street sports appeal to groups traditionally underrepresented in organized sport, including individuals with a short-term education and immigrant background (4). Consequently, street sport facilities are increasingly viewed as instruments for promoting inclusive and accessible spaces for physical activity and cultural expression (5). This trend aligns with a broader societal ambition to democratize sport and leisure opportunities.

Because many sports facilities in the Scandinavian countries are publicly funded, research has become concerned with the public value they create for citizens (6–8), often drawing on Mark Moore's theory (9, 10) of public value creation. The core premise of Moore's theory is that public institutions—or organizations receiving substantial public funding—should generate public value. The public value framework is beneficial when studying initiatives like street sport facilities, where substantial amounts of public funding are involved, but where value is not easily measured in economic terms, but instead emerges as social, cultural, ethical or democratic outcomes in a specific context. Studies show that although traditional sports facilities have a vision of creating value for all citizens, managers often end up focusing on sports associations (8). This means that other types of users, such as street sports practitioners, risk not being sufficiently accommodated. Many street sports practitioners are interested in gaining access to indoor facilities, but research in lifestyle and informal sports shows that it is a struggle to find space in traditional facilities for these types of activities and that these rarely meet their needs (11, 12). King and Church (12), for example, show how self-organized groups often struggle to access facilities designed by formal institutions—spaces that don't always align with the flexible and spontaneous nature of their activities. They also highlight that long-term access to suitable, low-threshold facilities is crucial for sustaining these communities. Building on this, Jeanes and colleagues (11) argue that many existing facilities are too structured and exclusive. According to Jeanes et al. (11), what's needed are more open, inclusive spaces that support spontaneous participation and community-building.

Despite growing academic interest in sports infrastructure, we know relatively little about facilities designed for emerging and informal movement cultures (13, 14), such as street sports. In Denmark, the NGO GAME has introduced the GAME Houses concept: hybrid indoor-outdoor facilities dedicated to street sport and urban culture. These spaces aim to democratize access, foster social inclusion, and support self-organized activities beyond traditional institutional frameworks. Municipalities and foundations have invested significantly in these facilities, reflecting confidence in their capacity to create public value.

This article investigates how the GAME House concept fosters public value by engaging local street sport communities excluded from conventional infrastructures. Specifically, we examine whether and how these facilities and their organizational structures accommodate the needs and aspirations of these groups. In doing so, the article contributes to understanding the societal value of street sport facilities and informs future planning and governance.

## The GAME house concept

The GAME House concept is developed by GAME, an NGO founded in Denmark in 2002 that focuses on promoting sports, physical activity, and social inclusion through street sports and urban culture nationally as well as internationally. They establish facilities for street sport and culture (e.g., graffiti art, DJing/playing and mixing music) and educate youth leaders as coaches and role models in street sport and civil society to make positive changes for themselves, for peers and for children.^1^ In 2010, GAME opened its first street sports facility, GAME House, in a former tram depot in central Copenhagen.^2^

The vision behind the GAME House was to create a street facility where children and young people from diverse social and ethnic backgrounds could engage in street sports in informal settings year-round. The facility was designed to be easily accessible, offering flexible activities in street sports and street culture, as well as opportunities for self-organized training and events. In addition to individual users, the 2.240 square meters was made to accommodate associations and informal groups engaged in street sports and culture, as well as primary schools and other institutions that wished to utilize the facilities. Based on specific organizational and architectural principles developed from the experiences gained at GAME House Copenhagen, three new GAME House houses were opened in the regional cities of Esbjerg, Viborg, and Aalborg between 2016 and 2018.

To investigate the public value creation of the GAME Houses concept, we will focus on these three facilities. These three facilities are of particular interest as they were conceived to provide purpose-built spaces for street sports practitioners, addressing the lack of infrastructure tailored to their activities, cultural practices, and organizational forms. The construction plans^3^ emphasize that local engagement is pivotal to the success of these facilities; they must be developed in response to locally articulated needs and aspirations, with community groups actively involved as co-creators in the everyday life of the venue.

## Theoretical framework

To investigate how the GAME House concept fosters public value by engaging local street sport communities, we will use Mark Moore's theory of public value (9, 10). His “Strategic Triangle” (see Figure 1) helps us understand how organizations with public funding can create and sustain value for society. Moore (9) argues that public welfare institutions ensure their survival by creating public value. Politicians reward these institutions for generating public value by allocating financial resources. To secure their long-term survival, welfare institutions must therefore appear legitimate, which they do, among other things, by documenting how they create public value for citizens and society (10).

**Figure 1:**
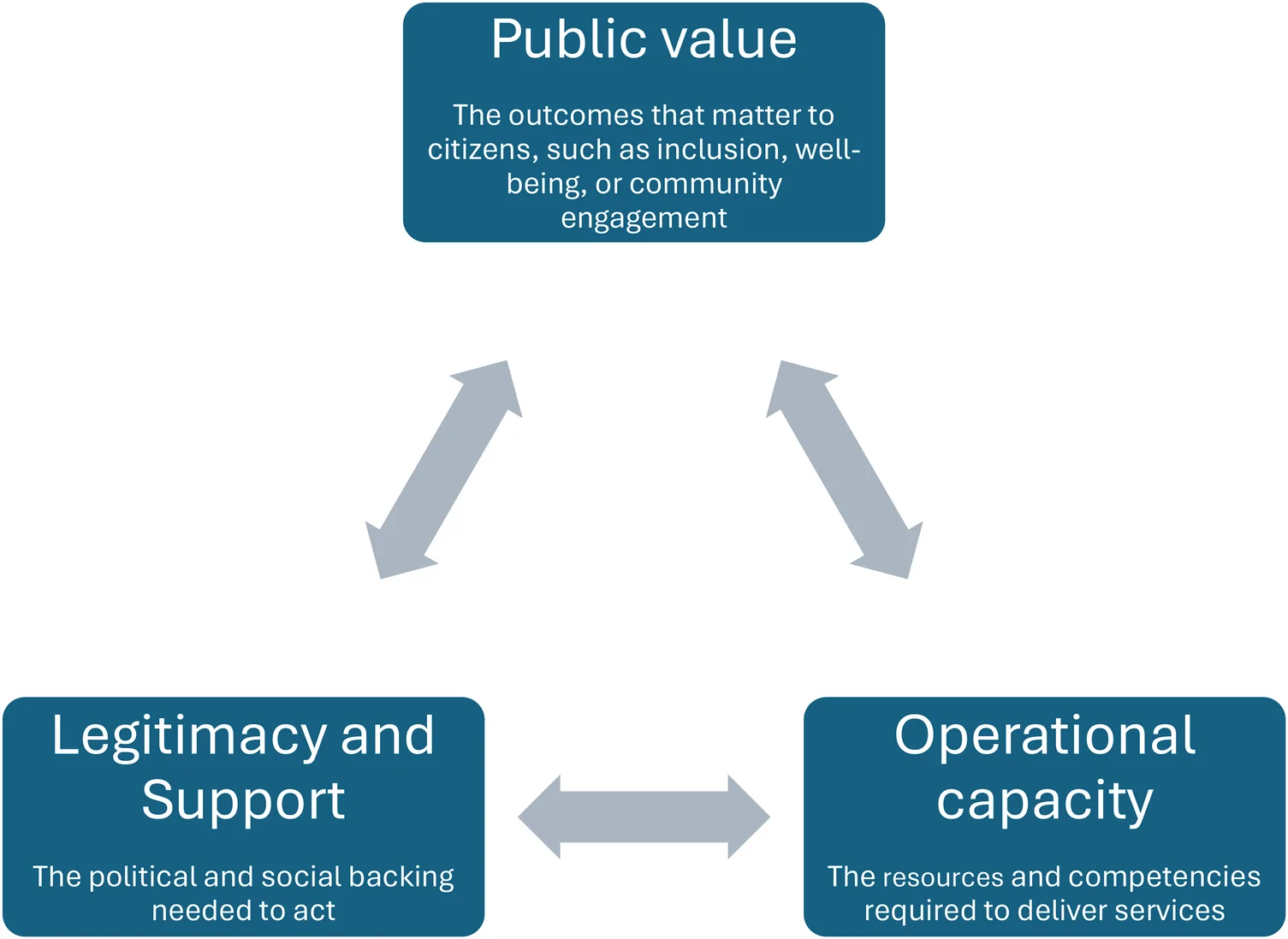
Moore's strategic triangle.

He summarizes the theory and the three key elements in the strategic triangle:

Operational capacity refers to the practical and organizational resources and competencies that an organization has at its disposal to deliver public value. This includes employee skills and expertise, organizational structures and leadership, physical infrastructure and technology, processes, systems, and workflows and capacity for innovation and adaptation. The idea is that all three key elements must be in balance. If one is missing, the initiative is unlikely to succeed in the long run (9, 10). In this study, we will use the strategic triangle as an analytical framework to explore the value of spatial support for street sports and their practitioners, as well as the conditions required to realize this value in practice.

Moore's theory of public value has been criticized for its lack of a precise definition of public value, which risks rendering the concept “all things to all people” (15, p. 408). However, as Alford and O'Flynn (16) note, public value should not be understood as “an absolute standard.” Rather, it is always embedded within a specific cultural and social context, shaped by the material, social, and ethical challenges that prevail in that setting (16). Although the concept of public value inherently implies value shared among multiple individuals, there is no fixed or clearly defined boundary. Moore instead emphasizes that a value can be public to a lesser or greater extent. In a 2014 paper, Moore explores the practice of public value accounting (17). He introduces the scope of public value through a matrix in which one axis represents *the valued object*, while the other represents *the arbiters of value* (see Figure 2). Both axes range from less public to more public (17).

**Figure 2:**
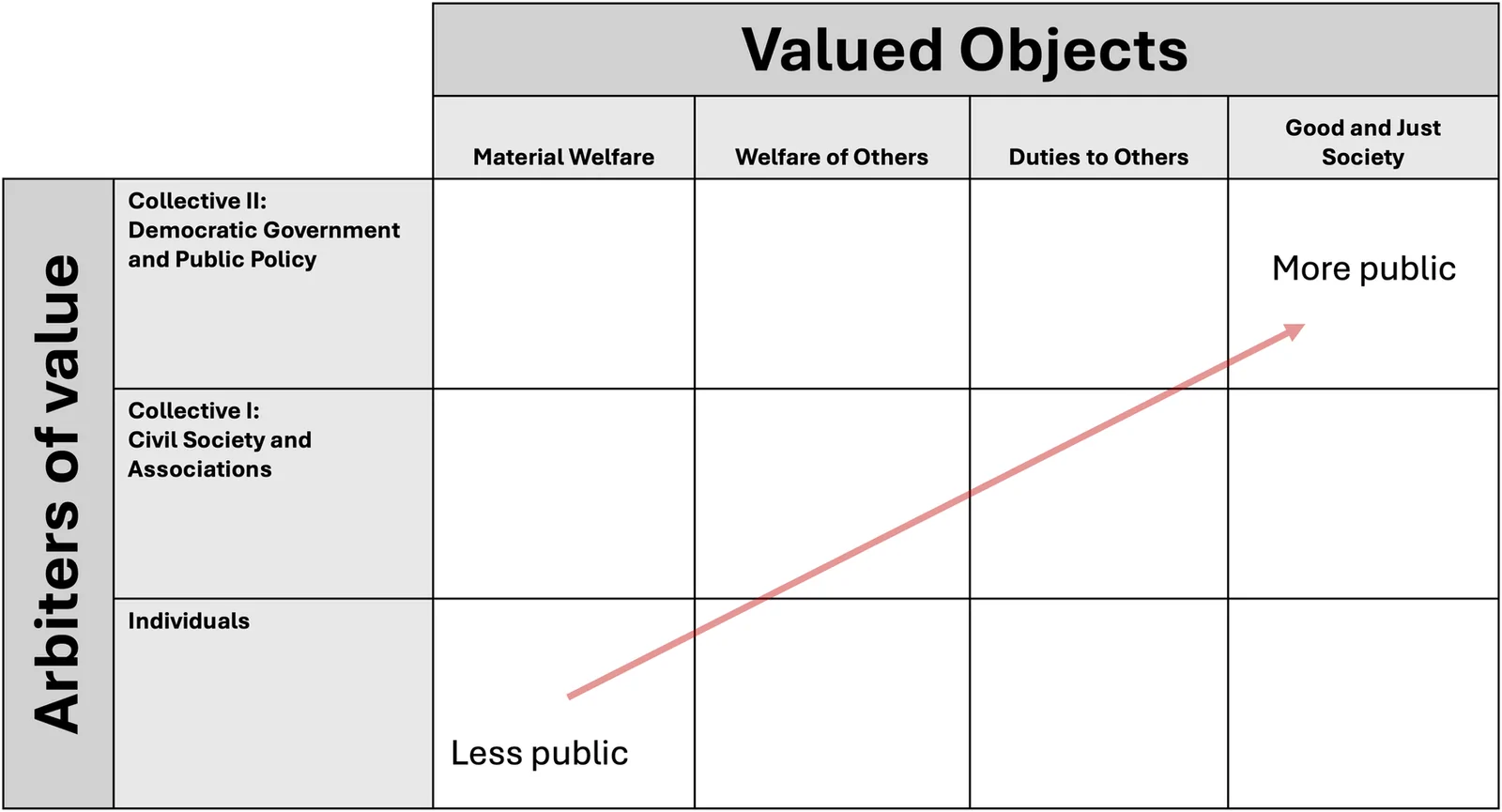
Authors' conceptual illustration of Moore's (2014) (17) framework of valued objects and arbiters of value.

The rows present the arbiters of value, which are the actors who evaluate what is good, right or just. The columns present the valued object, which is the domains in which these evaluations are made. The arbiters of value are divided into three levels, arranged from least to most public: Individuals, Collective I (Social Movements and Voluntary Associations) and Collective II (Democratic Government and Public Policy). The Valued Objects are divided into four categories, arranged from more private to more public concerns: Material Welfare, Welfare of Others, Duties of Others, and Concepts of a Good and Just Society. According to Moore (17), the valued objects range from a utilitarian perspective on material welfare and the welfare of others to a deontological perspective on duties to others and concepts of a good and just society.

While most studies in public values have focused on public policy and thereby the most public arbiters of value (Collective II), the primary arbiters of value in this study are members of *Collective I*, while the valued objects constitute the empirical focus of the analysis. Consistent with Moorés theory, however, public value also depends on broader public legitimacy and support. The analysis therefore incorporates the perspectives of municipal actors (*Collective II*), who not only provide legitimacy and institutional support for these values but also articulate how they contribute to broader public purposes. The GAME House vision to create facilities that attract and include those street sport groups marginalized in traditional sport associations and facilities might be a less public value from a utilitarian perspective, because it implies that we spend resources on a specific group of citizens. However, from a deontological perspective, it is highly public, because it is about creating a just society where everyone can be included in a sportive community that fits their life circumstances.

In the analysis, we analyze the facilities' ability to attract and foster meaningful collaborations with local street sports groups that do not fit within traditional sports or leisure infrastructures. The focus is on the value generated for different user groups, the evolution of legitimacy and support for the facilities, and whether operational capacity has been sufficient and effectively mobilized to create public value.

## Method

The study is a part of a broader study of the GAME houses in the municipalities of Esbjerg, Viborg and Aalborg.^4^ The analysis draws on document analysis as well as individual and focus group interviews with the included user groups of the facilities, along with managers, consultants and directors from the Culture and Leisure Departments in the three municipalities.

The document analysis consisted of a review of all project documents related to the establishment of the three GAME House facilities in Esbjerg, Viborg, and Aalborg. This included tender materials, construction plans, and operational agreements between GAME and the respective municipalities. The documents provided insight into the visions for the facilities as well as the specific agreements between the municipalities and the GAME House facilities. As such, they helped us to focus the interviews and provided background knowledge (18) to explore how the facilities are intended to support and develop the existing local street sport communities. These documents have formed the basis for the section that presents the GAME facilities in Esbjerg, Viborg, and Aalborg before the analysis.

Semi-structured telephone interviews (19) were conducted with 12 user groups. The 12 user groups were geographically distributed across the regions surrounding the three GAME Houses. Although telephone interviews have the limitation of not providing visual support for the dialogue—thus requiring more continuous clarification—they offer the advantage of not necessitating that the interviewer and the representatives be physically co-located (19). The representatives of the 12 user groups all preferred the telephone interview format. The interviews lasted between 1 and 1.5 h. Among other topics, they were asked to reflect on the activity-based communities they are part of, the nature of their collaboration with GAME House, their level of satisfaction with this partnership, and, importantly, the value that GAME House brings to their activities—both in terms of access to space, visibility, and opportunities for growth and engagement. We got in contact with the representatives through the three facility managers.

The analysis included all user groups that, at the time of the study, had formal agreements with their respective local GAME House facilities. These groups represent a broad and diverse spectrum of communities, characterized by varying organizational structures—ranging from entirely self-organized collectives to formal associations, as well as private actors and youth organizations. Table 1 provides an overview of the local user groups with formal agreements at the time of the study.

**Table 1:** Overview of the local street sports groups that have formed a formal collaboration with a GAME house.

Esbjerg		
Skate	Grassroots network	The representative owns the local skate shop Collabo, which functions as a central hub for the skating community in the area
Graffiti and street art	Grassroots network	The community associated with graffiti and street art is represented by Graffitiservice*,* a local graffiti art enterprise based in Esbjerg. The owner of the company plays a central role within the local graffiti scene and maintains connections with individuals active in the community.
Stylez Dance Academy	Commercial actor	A privately owned dance studio based in Aarhus, with a primary focus on the hip-hop genre.
Recovery Bulls	Social association	A non-profit organization providing diverse physical activity programs aimed at socially marginalized citizens, emphasizing social inclusion and community engagement.
Inline hockey	Organised community	Two youth teams affiliated with a local ice hockey club, where a coach takes the initiative to organize off-season training sessions in the form of roller hockey.
Viborg		
Viborg Parkour	Sports association	The association has three teams divided into age groups: 7–9 years, 10–12 years, and a 13–18 year's team, which at the time of the study was merged with the adult members of the association. All regular training sessions are held at House's facilities.
Dance by Heart	Commercial actor/sport association	Dance by Heart is a small company that has also established itself as an association to enable collaboration with House Viborg and gain access to the dancehall. The focus is fitness dance.
Viborg Youth School	Youth education programme	The municipal Youth School organizes weekly activities throughout Viborg Municipality, which children and young people can sign up for free of charge.
Aalborg		
Aalborg Street Workout	Sports association	A sports association offering activities in street workout and calisthenics. The association began as a self-organized community, which has since been formalized into an association.
Aalborg Climbing Club	Sports association	A sports association offering activities in climbing and bouldering.
Floortics Family	Grassroots network	A community centered around breakdance. The group functions as volunteers at GAME House Aalborg and conducts weekly training sessions.
DFUNK—Danish Refugee Council Youth	Social association	DFUNK is a voluntary youth organization with an association structure, working socially and politically for young people with refugee backgrounds. At GAME House Aalborg, DFUNK hosts its weekly food community, *Madmekka*, which focuses on cultural exchange, integration, and social interaction.
Recovery Bulls	Social association	A non-profit organization providing diverse physical activity programs aimed at socially marginalized citizens, emphasizing social inclusion and community engagement.

The three facility managers were interviewed face-to-face. The interviews took place in their respective GAME Houses after a guided tour and lasted between 1 and 2 h. Among other topics, they were asked about their collaboration with user associations and the municipality, their approaches to user involvement, their impact on the local area and specific user groups, and their relationship with the existing street sport community in the city. While different interview modes for the facility managers and the user groups may influence the nature of the interaction, all interviews generated rich qualitative accounts relevant to the analytical focus of the study.

The leisure consultants from the municipal departments for Culture and Leisure were focus-group interviewed online using Microsoft Teams. Two of the directors of the municipal departments for Culture and Leisure were individually online interviewed using Microsoft Teams, while one was individually telephone interviewed. While the group interview with the consultants provided an opportunity for them to share their experiences collectively—and enabled us to identify similarities and differences across the three municipalities—the individual interviews with the directors allowed for a more in-depth exploration of each municipality's specific experiences with the GAME House facilities. Both the consultants and the directors were asked about their knowledge of street sport, their previous experiences with supporting it locally, the nature of their collaboration with GAME House, how the facilities interact with the established sports infrastructure in the municipality, and the impact GAME House has had on children and young people, the local community, and specific user groups. Both the consultants and the directors preferred online interviews and had positive experiences with computer-mediated communication following the COVID-19 pandemic. The online interview format, in contrast to telephone interviews, had the advantage of being visually supported, which facilitated a more effective dialogue between interviewer and interviewee (19).

All interviews were audio-recorded and transcribed. Data management complied with current GDPR legislation and was approved by the Research Innovation Office (RIO) at the University of Southern Denmark (no. 10.159).

## Presentations of the GAME houses in Esbjerg, Viborg and Aalborg

The three GAME Houses in Esbjerg, Viborg, and Aalborg were established between 2016 and 2018. See Table 2 for a overview of the three GAME Houses. Each is in an obsolete industrial building that has been transformed and adapted to accommodate street sports and urban culture. The underlying premise is that such architecture embodies both functional and identity-forming qualities for participants and the street culture; accordingly, the specific architectural characteristics of the buildings have largely been preserved. All three facilities are centrally located and easily accessible by public transportation. GAME House Esbjerg and GAME House Viborg are located within 1 km of the central railway station and bus terminal. GAME House Aalborg is situated in a newly developed urban residential area 1.8 km from the central railway station and bus terminal. However, there is a bus stop situated only 200 meters from the facility. GAME House Viborg is located in an urban renewal area bordering the socially vulnerable residential neighbourhood of Ellekonebakken, while GAME House Esbjerg is located approximately 3 km from the nearest socially disadvantaged residential area. All three facilities feature both outdoor and indoor spaces. The outdoor areas are publicly accessible, whereas access to the indoor facilities requires either a membership or a day pass. Unlike many other sports and cultural facilities, GAME House almost always remains open—even on traditional closing days, such as Christmas Day and Easter. Users can use the facilities for self-organized training, join open classes offered by the house or join closed classes offered by the different local user groups and associations.

**Table 2:** Overview of the three GAME houses in Esbjerg, Viborg, and Aalborg.

	Esbjerg	Viborg	Aalborg
	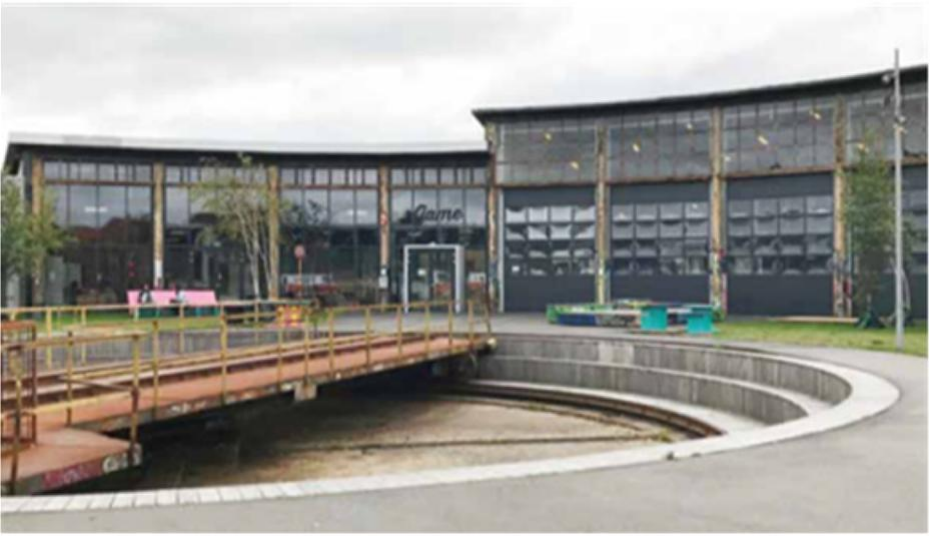	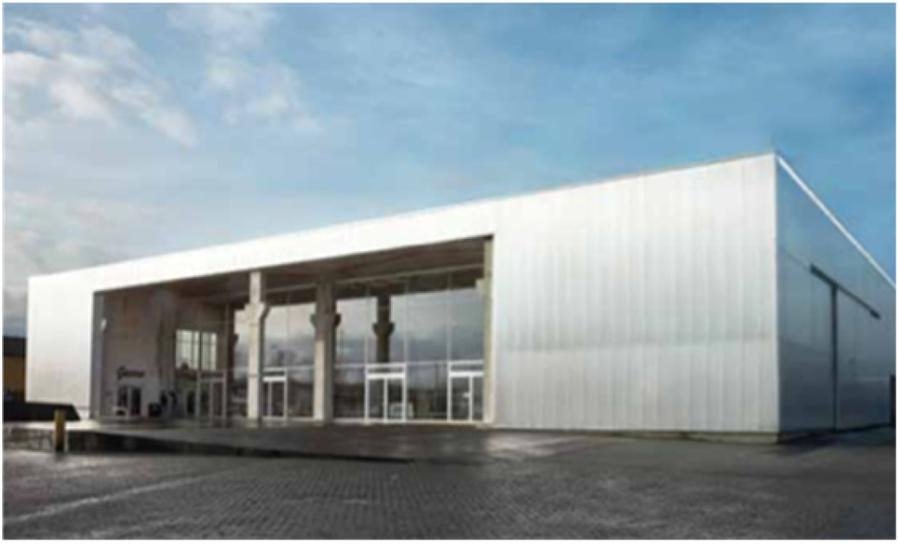	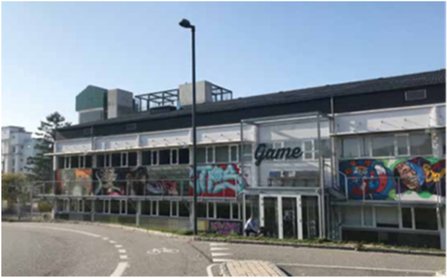
Opening	January 2016	March 2018	February 2018
Total cost	3.350.000EUR	4.490.000EUR	3.350.000EUR
Size	2,800 m^2^ (indoor) and 1,500 m² (outdoor)	3,180 m^2^ (indoor) and 2,000 m² (outdoor)	2,500 m² (indoor) and 500 m² (outdoor)
Facilities	Scootering/Kick scooter	Street basketball	Street basketball
	Skateboarding	Street soccer	Street soccer
	Street basketball	Bouldering	Calisthenics// Street workout
	Parkour	Parkour	Parkour
	Street soccer	Trial/Bike trial	Bouldering/Climbing
	Table tennis/Ping pong	Scootering/Kick scooter	Slackline
	Dance	Skateboarding	Floorball/Street hockey
	Panna	Dance	Dance
	Roller hockey	Roller skating	Panna
	Roller skating	Street art/Graffiti	Slackline
	Street art/Graffiti	Visual workshop	Street art/Graffiti
	Calisthenics/Street workout	Calisthenics/Street workout	Table tennis
	Floorball	Table tennis	Table football/Foosball
	Rap/DJing/Music production	Table football/Foosball	Rap/DJing/Music production
	Yoga	Panna	Yoga
	Lounge/Chill-out area	Yoga	Lounge/Chill-out area
		Rap/DJing/Music production	
		Workshops (Wood and Metal)	
		Lounge/Chill-out area	
Training program	(Autumn 2017)	(Autumn 2018)	(Autumn 2018)
	Scootering	Skateboarding	Bouldering
	Parkour	Parkour	Street workout
	Breakdance	Hip-hop	Breakdance
	Street basketball	Street soccer	Slackline
		Street basketball	Street basketball
		GAME Girl Zone (activities targeted at girls)	Parkour
		Dance	Hip-hop
		Wood and Metal Workshop	

Each GAME House is organized as a local branch under the umbrella of GAME Denmark. The operational framework for these facilities is established through long-term agreements (10–12 years) between the national association GAME Denmark and the respective municipal authorities. Municipalities provide an annual operating subsidy, which does not cover potential deficits. To ensure the continuous operation and development of activities, GAME relies on additional fundraising through foundations and internal revenue.

The key actors within each GAME House include *a local staff*, *user associations*, which use the facilities as platforms for their own programs while contributing to the overall life of the GAME House through activities open to all members; and *user councils*, which are to ensure user and association influence and serve as a voice for those wishing to contribute beyond their own participation. The user councils constitute themselves, establish their own rules of procedure, and meet at least quarterly. Councils are planned to be composed of one member from each user association (appointed by the respective association), two local users elected by the facility's members, and two members appointed by GAME and the daily manager. The staff is responsible for daily operations, coordination of local volunteers, and support of the local user council.

The organization of activities within GAME Houses is planned in close collaboration between the local staff and the user associations, which are expected to offer their programs within the facilities. For each user association, a cooperation agreement is to be established with the daily manager, based on the association's competencies and capacity. Local volunteers are also intended to be involved in organizing weekly training sessions in street basketball, street soccer, street dance, and parkour, as well as a variety of events and workshops, building on the extensive experience and success with volunteers at the GAME House in Copenhagen.

## Analysis

In the analysis, we investigate how the GAME House concept fosters public value by engaging local street sport communities excluded from conventional infrastructures. We begin by showing an overview of the local street sports groups that have formed a formal collaboration with a GAME House (Table 1). After that, we explore the value generated for different user groups, the evolution of legitimacy and support for the facilities, and whether operational capacity has been sufficient and effectively mobilized in relation to the intended public value.

## The value creation for the included user groups

The analysis of the qualitative interviews revealed that access to dedicated facilities was valuable for the different street sport communities in three distinct ways: 1) the communities desired but lacked any other facilities meeting their practical and cultural needs, 2) they considered dedicated facilities essential for strengthening their activity-based community, and lastly, 3) the communities found the dedicated facilities important in recognizing their activities as valuable as traditional sports. In what follows, we outline these three ways in which the facilities emerge as significant for the different street sports and their communities.

### Meeting practical and cultural needs

The analysis reveals that a central contribution of GAME House lies in offering facilities tailored to the needs of diverse street sport communities, addressing the widespread lack of secure and appropriate spaces for practice. For example, before the opening of GAME House Viborg, the local parkour association had been facing ongoing challenges. For six years, they were moved between temporary facilities provided by the municipality:

“Before, we were in a hall that wasn't heated, and the toilets weren't cleaned […] and then we were in the youth school's premises for a couple of months, and after that, we were without any facilities for a while before GAME opened.”

(Rep., Parkour Viborg)

Similar experiences were shared by other groups. Before the establishment of GAME House Aalborg, the local street workout community had to rely on outdoor public equipment or commercial gyms—options that were either weather-dependent or financially restrictive. Although these groups identify with a street-based culture, they expressed a strong need for indoor facilities that support focus, group activity, and consistency in their practice.

The local street roller hockey team in Esbjerg explains:

“They [Game House] have the perfect setup for what we need. So in that sense, it means a lot. Otherwise, we'd have to play on a closed-off street or something like that—and we can do that too. But then we'd have to move the goals every time a car comes by. Just like we did back in the day. So it's really great that we can do it under such proper conditions.”

(Rep. Street roller hockey, Esbjerg)

Although street sports traditionally take place in the raw and unstructured spaces of the city, many activities require specific spatial conditions to thrive (5). In the case of street ballgames, the quality of the asphalt surface, the design of hoops or goals, and the surrounding architectural context significantly shape users' experiences and their attraction to the site. As one older user of GAME House Aalborg describes:

“Down by the harbor, the facilities are good, but it's the small details that matter—like whether the hoops are good  … There's no street vibe down there, and when it gets wet, it becomes really slippery. It can also get quite windy because it's right by the water … so there are several small things.”

Across all three GAME Houses, user groups consistently highlight the perfectly smooth asphalt surface as a defining quality. Besides offering tailored facilities that enable them to train consistently and safely, and meeting their spatial needs, many of the groups also highlighted the cultural atmosphere as important to them.

Breakdance originated in the boroughs of New York during the 1970s as the dance element of the broader hip hop culture, which developed as a lifestyle and a form of social activism in urban spaces (20). Although breakdancing is culturally still inextricably linked to urban spaces, most of the training and organization of events takes place in private dance studios in Denmark. But, as the breakdancers at GAME House Aalborg explained, the GAME House environment is a better cultural fit than the traditional private dance studios.

“The breakdance culture is very free-spirited, it comes from the streets, and sometimes it simply doesn't fit into a formal dance studio—I felt that myself in the end, in terms of my own motivation. I knew that a GAME House was about to open in Aalborg, and I thought: That's where we need to go. That's exactly the kind of environment we're missing…”

(Rep. breakdancing group, Aalborg).

A consistent architectural element across the GAME Houses is the perimeter, composed of openable gate panels with large windows (see Figure 3). These can be opened when the weather permits, allowing users to feel as though they are outdoors and creating a synergy between indoor and outdoor environments. This design helps preserve the street-cultural atmosphere that is central to the identity of street sports.

**Figure 3:**
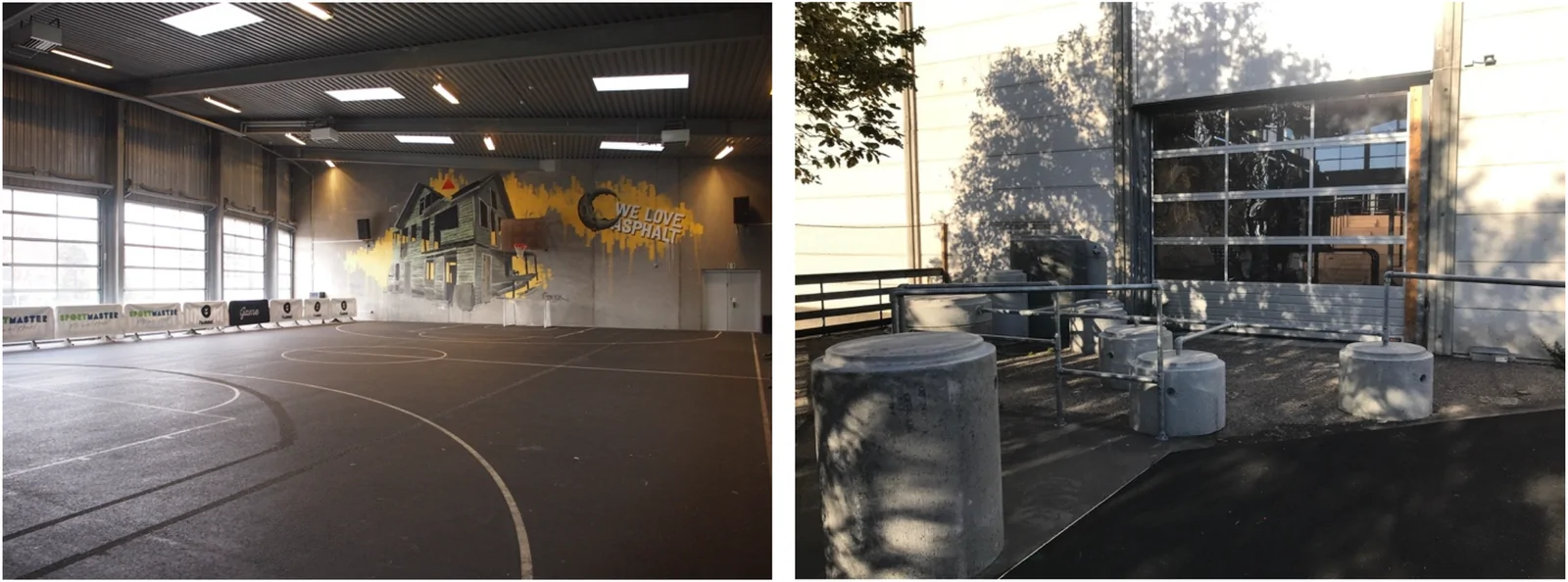
Openable gate panels in GAME house esbjerg (left) and Aalborg (right) enable direct interaction between indoor activities and the surrounding public space. Source: Author's own photograph.

### Strengthening communities

The analysis also reveals that providing these tailored facilities significantly impacts community development and visibility. GAME House has served as an important platform for showcasing the activities of user groups. The representative for skateboarding in Esbjerg describes how the popularity of skateboarding in Denmark has fluctuated since the 1970s. According to him, facilities such as GAME House help ensure a more stable, local interest in skateboarding.

“…some years it's more popular than others. But when you have a facility like GAME House, it creates an environment that is lasting, and you can see … We ran this skate school project last year, where we had different public school classes participate. They could choose it as an elective subject, and we were two instructors teaching them there. You can really see that this whole generation—they're just there all the time. They've become a part of it. They're not there because it's summer, or because it's trendy, or because they happen to know someone who has a skateboard or something like that. They're there because they are skaters now.”

(Rep. skateboarding, Esbjerg)

As the representative describes, the facilities at GAME House have enabled the user association to conduct programs with public school classes, which he believes has been crucial in encouraging more children and young people to engage in skateboarding activities. The facilities give kids a chance to discover street sport—and to see that it's an option for them. In Viborg, the representative for Viborg Parkour also explains that becoming part of GAME House Viborg has significantly increased the exposure of their activities. This happens through visiting users, but especially through visiting schools and institutions.

“Children and adults simply know that if you want to do parkour in Viborg, it's at House, and that has meant that the groups have grown from perhaps 7–13 participants to currently around 20 participants.”

(Rep. parkour, Viborg)

This highlights how the GAME Houses both strengthen the local community and, at the same time, support the vision of making street sports more accessible as a public value.

### Recognizing street sport and street cultures

Another value is that the GAME Houses help legitimize and normalize street-based activities that are often misunderstood or marginalized. In Esbjerg, the representative for skateboarding describes how GAME House played a crucial role in creating and supporting a growing skate and youth community in the city. He views Esbjerg as a town characterized by a traditional sports culture, with a strong focus on elite sports. He points out that many residents of Esbjerg are therefore unfamiliar with street sports and may even hold a negative perception of the children and young people who skateboard. The negative perception of children and young people who skateboard is not unique to Esbjerg but is richly documented in academic literature. Skateboarders have generally been viewed as marginalized youth who disrupt the capitalist and functional order of the modern city through their transversal and noisy movements (21–24). GAME House Esbjerg was thus an important factor in raising awareness of skateboarding, as well as improving the reputation of skaters:

“Esbjerg is very old school. It's mostly football, handball, ice hockey—those elite sports that dominate the scene. And I think that's because the people of Esbjerg simply haven’t been introduced to anything else. So, skateboarding and all the street sports have struggled to shake off the image of ‘They’re just troublemakers, those skaters. They wear baggy pants and all that.’ That was something from the ‘90s. But it doesn’t exist anymore. It has taken longer to shed that image here than in the rest of the country. That's why we really need a place where you can see, ‘Something is really happening here,’ with all kinds of different people … those guys in their 40s, 42s, and 44s, bringing their kids along, and there are very young people, and all sorts of different people coming.”

(Rep. skateboarding, Esbjerg)

For some user groups, GAME House have had a very strong impact on their legitimacy in the broader public. The establishment of GAME House Esbjerg has had a significant impact on the community surrounding graffiti and street art, as it has provided them with a legal and public social meeting place. Being able to meet and paint together is important not only for the social community but also for the individual development of the artists. The representative for graffiti and street art describes the culture as divided into two groups: those who paint for the sake of art, and those who paint for the thrill. The first group values the cultural history of graffiti and keeps up with how the art form develops nationally and internationally. While this first group is characterized by paintings (so-called pieces), the second group is characterized by scribbles or names (so-called tags). The representative emphasizes that a place like GAME House cannot attract the latter group, as the illegal aspect is central to them. Nevertheless, GAME House Esbjerg has had a preventive effect, since many of those who paint for the sake of art previously struggled to find legal facilities. As he explains:

“But I could feel … before, many had difficulty finding legal places. They might occasionally paint in a tunnel or something like that because they felt it was the most harmless option if they had to. But now they have switched to just painting over there. They now have all the opportunities. So it has had a preventive effect. It's not like back when we started. At that time, there wasn’t a legal wall. We had to do it illegally. But now you don’t have to be a criminal just to paint graffiti. You are allowed to do it and practice it as a legitimate art form.”

(Rep. graffiti and street art, Esbjerg).

In addition to providing a legal wall and thus having a preventive effect, the representative describes how the facility has helped raise greater awareness and knowledge of graffiti and street art in the public consciousness. He explains that they are seeing more young people come by and want to try painting. Previously, they had no legal opportunity to do so. Because the wall is located along a walkway, curious passersby encounter it daily (see Figure 4).

**Figure 4:**
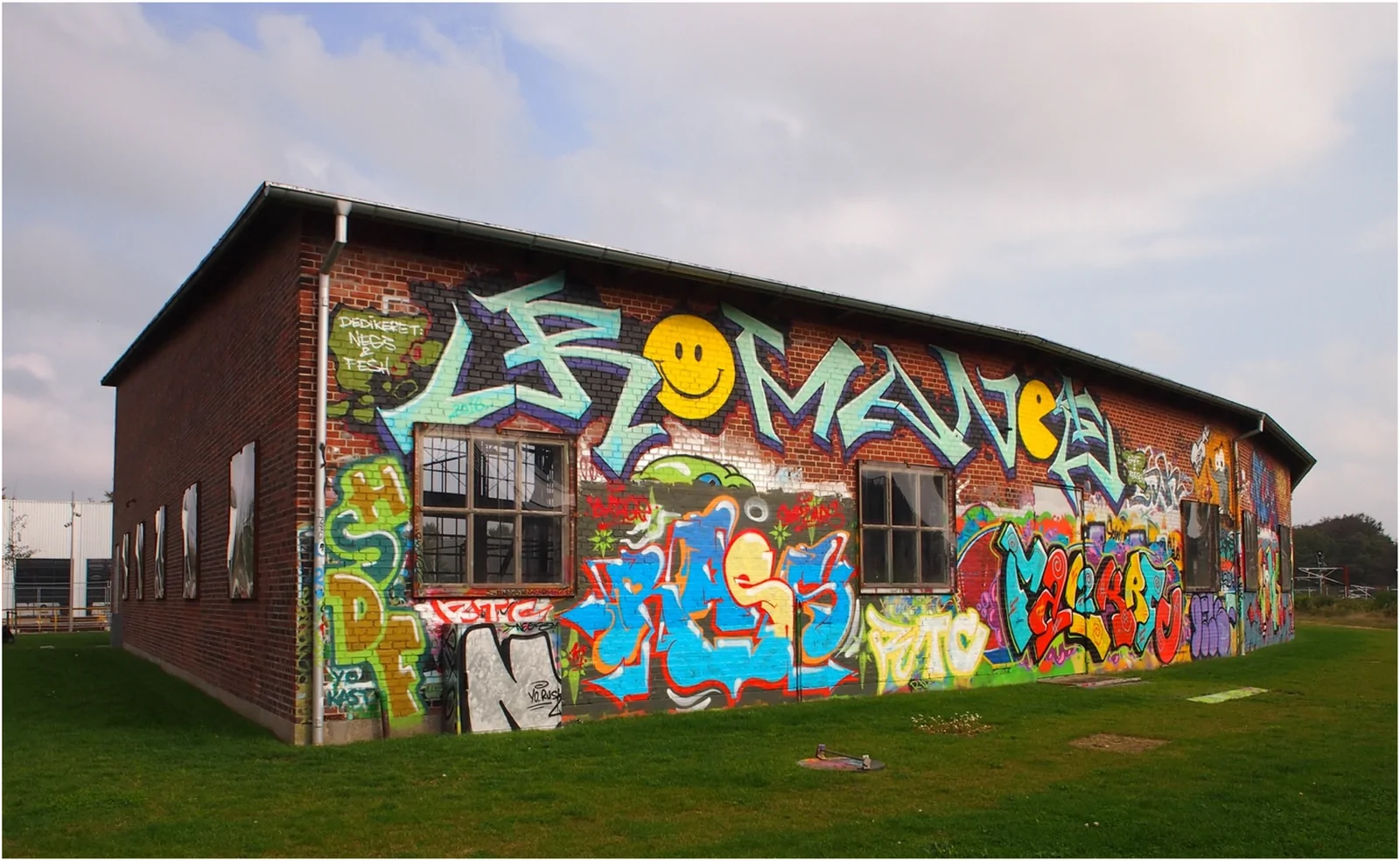
The legal wall for graffiti and street art in GAME house esbjerg. Source: Author's own photograph.

The representative describes how they often experience people stopping to ask questions about their paintings and practice. These individuals are interested in knowing whether the artists are happy to have the space, what they do, why they write the things they do, and how they come up with their ideas. The opportunity to meet active participants and ask curious questions helps foster greater understanding of the community and the distinction between graffiti and street art as, respectively, art and vandalism.

“Graffiti artists have probably always had somewhat of a mixed reputation, as it is associated with something criminal in some way. So when people see others walking their dogs up there, or young people cycling past, they realize that these are kind and pleasant people of all ages who are painting (…) They get a face to put to the people behind the image of someone wearing a hoodie in a tunnel—they are just ordinary people who want to express themselves creatively.”

(Rep. graffiti and street art, Esbjerg).

Thus, the GAME House has contributed to legitimizing the graffiti and street art community in Esbjerg. As the representative of the graffiti and street art in Esbjerg mentions, there have been some concerns about providing physical facilities for graffiti and street art within the GAME House. While he understands these concerns, he believes that establishing legal facilities is precisely part of the solution to many of these issues.

These three main findings show how the facilities support and foster the growth of local street sports and street culture by giving them access to facilities that meet their practical and cultural needs, strengthen their community, and, in that way, recognize their activities as valuable and wanted. The practical and cultural needs expressed by the user groups can, in line with Moore's matrix of public value (17), be understood primarily as concerns related to material welfare—that is, access to adequate facilities, safe and functional spaces, and the infrastructural conditions necessary for sustaining their practice communities. Yet the value they attribute to GAME House extends beyond these utilitarian considerations. When they emphasize the importance of recognition, belonging, and the cultivation of inclusive practice communities around activities such as skateboarding or street art, their reflections point toward broader conceptions of a good and just society**.** In this sense, the spatial support offered by GAME House generates public value on two levels: it meets concrete, practice-oriented needs while simultaneously contributing to normative ideals about fairness, inclusion, and the right to participate in urban cultural life. The support, therefore, carries significance from both a utilitarian perspective—focused on welfare and functionality—and a deontological perspective—concerned with duties, rights, and the moral conditions of a just community.

We will now turn to the analysis of the conditions required to realize this in practice.

## The operational capacity

In all three GAME houses, the local staff consist of two full-time employees: A daily manager who is responsible for management, administration, and operations of the GAME House, and a project coordinator who handles bookings, events, and practical everyday tasks. As part of the local staff, all three facilities also employ junior coordinators. These are young staff members—often students—who support the daily operation of the facilities and facilitate activities, events, and user engagement, acting as accessible role models within local street sport and street culture communities. Building on the extensive experience with volunteer engagement at the GAME House in Copenhagen, local volunteers were expected to play a key role in organizing weekly street sport training sessions across all sites. The daily managers of all three GAME Houses highlighted volunteers as essential to the implementation of training sessions, activities, events, and programs within the houses.

“It's a good thing we have the Playmakers [volunteers between 16 and 25 years], because they help by setting up regular weekly activities or supporting our events. But it's not just that—the Playmakers also help ensure that the young people in the house get involved. Sometimes we have users who come here without a group of friends, and they don’t always have someone to do things with. In those cases, we or the Playmakers figure out something for them to do. There are times when the Playmakers step in and, for example, say: ‘Let's gather all the girls and go outside to jump rope or come up with something else,’ and that's just really great.”

(The Facility Manager of GAME House Viborg)

However, while GAME House Viborg and GAME House Aalborg were able to recruit a substantial number of local volunteers, recruitment was considerably more challenging in GAME House Esbjerg. The daily manager attributed this challenge to a combination of youth outmigration from the city and limited organizational capacity to conduct targeted volunteer recruitment efforts.

According to the building program and operational agreements, street sport sessions were intended to be offered by the affiliated local user groups. In practice, however, it has not been possible to involve user groups in all activities. This also suggests that certain facilities have been built because local children and young people were assumed to want them, even though there has not been a real user base large enough to sustain organized activities there. In some cases, the centers have been required to establish collaborations with user groups that were not locally based or use paid instructors to activate the facilities. This is, for example, the case with Stylez Dance Academy at GAME House Esbjerg, which was a private dance school based in Aarhus. In relation to parkour, GAME House Esbjerg likewise found it necessary to employ two paid instructors, although these were locally based. The two young men engaged in parkour practice but were not embedded in any organized group related to their activity. According to their own account, they were the only individuals practicing parkour in the area. They expressed no interest in establishing a team targeting their own peer group; however, they indicated a willingness to set up children's teams on a remunerated basis. In all three GAME Houses, the daily managers noted the challenge of balancing the involvement of volunteers and user groups with the use of paid instructors.

Table 1 shows an overview of the user groups with a formal collaboration with one of the three GAME houses. They represent both grassroots networks, sport organizations, social associations and commercial actors and a wide variety of activities, from food making to breakdance. The different types of user groups challenged the planned structured collaboration model. The idea was that both informal and organized user groups have entered a collaboration with their local GAME House, could use the facility for closed association hours in exchange for providing instructors for an agreed number of activity hours (free open training sessions for the house's users).

Through the interviews, the managers explained that some groups were too small or loosely organized, and others preferred open formats that welcomed everyone—regardless of affiliation. The idea of separating open and closed training didn't align with some of the user groups' inclusive spirit of their activities and communities. Therefore, the managers found alternative ways to collaborate. Some groups get exclusive access outside opening hours in return for helping with events. Others offer open sessions voluntarily. These flexible arrangements are tailored to each group's resources and needs. As one of the facility managers put it:

“It's about local adaptation. You simply must figure out what works locally. I don't think you can create a one-size-fits-all concept. Denmark may not be large geographically, but culturally, it's big.”

(Facility Manager, Esbjerg)

This highlights a crucial point: the success and value of these collaborations lie in their adaptability. By acknowledging the diverse needs and capacities of different groups and shaping agreements accordingly, the partnerships become both meaningful and sustainable. What could not, however, be accommodated was the requirement that user groups share the facilities and are active together under the same roof (see Figure 5). Originally, the Viborg Cycle Trial Club was involved in the development phase of the GAME House, but withdrew when they felt they could not maintain the same club atmosphere while sharing facilities with other activities. The Trial Club also had less need for the facilities than some of the other user groups, as they already had access to a hall near the GAME House. Although the Trial Club chose to withdraw as a formal user group, its members occasionally make use of the facilities for their training.

**Figure 5:**
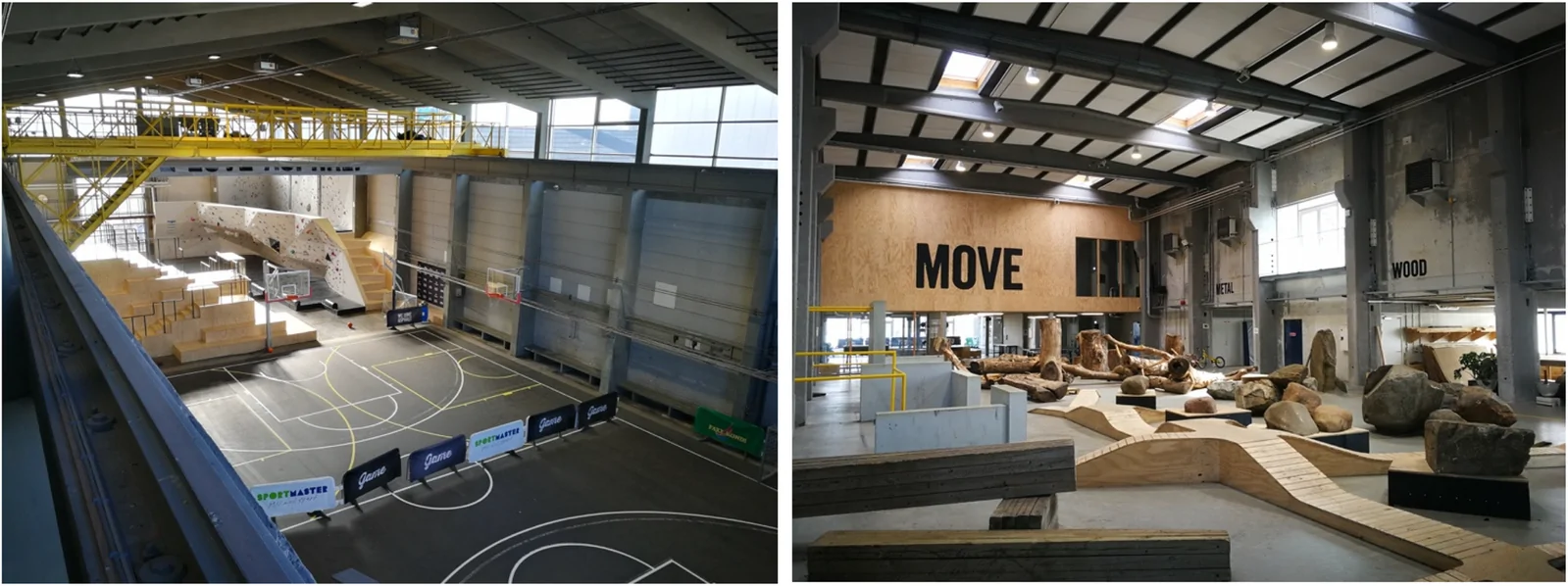
Indoor activity areas in the GAME houses in Aalborg (left) and in viborg (right). Source: Author's own photograph.

The original idea also included a formal user council, with the aim of strengthening local ownership and engagement. But in practice, a user council was never established in any of the three houses. In the qualitative interviews, the managers expressed how a challenge in creating the user council was the diversity of user groups. The managers found that many were focused on their own discipline—like skateboarding or dance—and weren't particularly interested in broader collaboration. Managers found it more meaningful to engage with user groups individually, and due to limited time and resources, formal user councils were never established. In 2018, two years after the opening of GAME House Esbjerg, GAME Esbjerg applied to the municipality to revise the description of user council involvement in the house's operational agreement. Drawing on experiences from the GAME Houses in Copenhagen and Esbjerg, the intention was to replace closed user councils with open workshops accessible to all users. The municipality of Esbjerg approved this change in the operational agreement.

Instead of the regular meetings in user councils, collaboration therefore happens informally, and there is an *ad hoc* dialogue between the different user groups and the daily managers in the three GAME houses. As one of the facility managers explains:

“Everything that is realistic for us to act on, we do (…) we listen, and we try to respond to everything we can. That includes everything from open training sessions to events, the content of events (…), so I feel that in that regard, we're doing quite well. We're really good at listening to what they have to say.”

While this clearly goes against the participatory governance model upon which the house is founded, the representatives are, in the three GAME Houses in general, happy about this collaboration mode:

“It's a very open environment, and the facility manager plays a big role in that. She's incredibly welcoming and approachable, and she always asks for our input—because she knows we're the experts. Whether it's something we need or when they're planning events like the Friday Jams, she listens.”

(Rep. skate, Esbjerg)

There's mutual respect, as this quote from the representative from the skate community explains, but it also places high demands on the facility managers, who must be present, attentive, and responsive.

## Legitimacy and support

According to Moore's theory (9, 10), the value GAME House creates depends on the legitimacy and support it receives, both bottom-up from local communities and top-down from the municipality. We will begin by outlining the legitimacy bottom-up from the local communities, and after that, the legitimacy top-down from the municipality.

### Legitimacy bottom-up

The analysis shows strong support from the various user groups. This strong level of support is clearly linked to the extent to which the facilities meet both practical and cultural needs. Although the establishment phase is not included in the present analysis, the high degree of needs fulfilment and support suggests that the extensive user involvement during both the design and establishment phases has been successful. Several workshops were conducted in which local users were invited to provide input on questions related to the design of the facilities. The few exceptions that do exist, however, underscore the importance of recognizing user groups as experts in their own disciplines to foster support. While professional architects were involved in designing the facilities, the parkour practitioners and the skate group from Esbjerg felt their local knowledge was overlooked:

“For example, when they built the skate facilities, GAME had hired a kind of skate-facility consultant to help say, ‘Yes, that's approved,’ or ‘That'll work,’ and so on. Instead of using some local people … even though we know some local experts have been doing this for 30 or 25 years, or however long these skate folks have been at it. Instead of involving locals in that process. So, I think they could be even more local in their engagement.”

(Rep. skate, Esbjerg)

The representative explains that all local users were invited to idea workshops where they could share a wide range of wishes for the facility. Some nice architectural drawings were created based on these wishes, but then the budget was suddenly reduced, which meant that many of the original ideas were not included in the final designs. This shows that the intention to involve local users was genuinely present, yet other factors ultimately limited their influence, leaving them with the sense that their input did not shape the outcome in any meaningful way.^5^ In Esbjerg, the parkour setup was also criticized for reflecting Copenhagen-style practice, which didn't match how parkour is done locally. The analysis makes it clear: street sports may be global, but they're practiced in locally specific ways, as the emerging research in the *glocalization* of lifestyle sports has also shown (2, 25–27). Glocalization is a concept developed by Robertson (28), describing the simultaneous tendencies of universality (globalizing) and particularity (localizing). As an activity, parkour is characterized by universal ways of articulating the practice, yet its concrete expression may vary depending on cultural and physical context, as well as sport legislation. As our analysis shows, that needs to be reflected in designing facilities for street sports—not just to ensure relevance, but to build bottom-up legitimacy and ownership.

### Legitimacy top-down

From the municipal level (top-down), legitimacy and support have been strong since the opening of the GAME Houses in Esbjerg, Viborg and Aalborg. Based on qualitative interviews with consultants and directors of the departments of sport and leisure in the three municipalities, they are very satisfied with the GAME Houses, which offer flexible and inclusive spaces for self-organized sport and leisure. This is further reflected in the fact that Aalborg Municipality, in 2019—only one year after the facility opened—increased the annual operating subsidy by DKK 250,000 for three years, following its success in attracting a high number of visitors. In 2023, the additional operating funding was extended for another three years.

Interestingly, the GAME House could have been seen as a competitor to traditional sports associations. But the municipalities actively worked to avoid that tension. Their strategy was to present the GAME House not as a rival, but as an inspiration—something that could broaden the local sports landscape, as this quote from a Head of a municipal department of sport and leisure shows:

We've held many of the local council for non-formal education and voluntary associations meetings at GAME House to send a clear signal: the organized sports community stands behind this initiative. From the beginning, we wanted to avoid an “us versus them” dynamic. Instead, we said: “GAME is part of the sports community”

The municipal level legitimacy and support for the GAME Houses is, however, closely tied to GAMÉs national status and reputation. GAME's work has contributed significantly to establishing street sports as a recognized concept within municipal culture and leisure administrations, among major sports organizations, and in the broader public discourse (5, 29). Since 2002, GAME has been a leading organization in Denmark working to make street sports and street culture accessible to a broader group of children and young people. Among its initiatives, GAME runs the program *Your GAME*, which uses street sports and street culture as a foundation to create activities for children and young people in disadvantaged residential areas in Denmark. The program has received considerable positive attention, and since 2002, GAME has been able to develop and implement new projects and initiatives—both nationally and internationally—based on external funding.

The legitimacy of GAME has been further strengthened by broader societal changes. Street sports have received increasing attention from municipalities and major national sports organizations, as young people are more likely than before to engage in self-organized physical activity. Consequently, there is growing recognition of the importance of active urban spaces for local community development, health, and well-being. Today, the vast majority of municipalities have one or more facilities dedicated to parkour, skateboarding, or street basketball.^6^ As a result, the GAME House concept has, from its inception, enjoyed a high degree of legitimacy both within the three participating municipalities and among the funding foundations. For the construction of the facilities, the municipalities have, in total, received external funding from major Danish foundations amounting to approximately EUR 1.9–2.6 million, while municipal co-financing has ranged between approximately EUR 0.7–1.8 million. This combination of external funding and substantial municipal investment reflects strong institutional support for the initiative and underscores the perceived value and legitimacy of street sport facilities as a tool for social and community development

Since opening**,** support and legitimacy from the municipality have only grown, as stated by both the municipal consultants and the heads of the municipal departments for Culture and Leisure. The GAME Houses are now seen as hubs for collaboration and knowledge-sharing. As one of the municipal consultants put it:

GAME didn't just say, “Well, now we have this building, and it's all about us.” On the contrary, they've shown a real desire to play a role in the broader street culture and in the municipality. GAME and House have helped us become better as a municipality—better at providing facilities and activities we wouldn't otherwise have been able to offer. In that sense, they've truly enhanced the quality of our work.

As the quote shows, the legitimacy and support are based on the fact that the GAME Houses help the municipality work in a more qualified manner to provide more flexible and inclusive facilities for sport. The consultants and heads of the Municipal Departments for Culture and Leisure also interviewed and unanimously emphasized that the consolidation of various activity offerings under one roof, combined with the distinctive organizational model and physical environment, helps attract and engage children and young people who would otherwise not have shown interest in street sports or street culture.

By placing it within a structured framework—by bringing things together—it is my impression that it draws in the curious. In other words, you reach new customer groups, or new groups of children and young people, who might not otherwise have developed an interest in this.

(Consultant in a Municipal Department for Culture and Leisure)

In this way, the various street sports and street-cultural activities have been made more accessible than in their original, self-organized forms. The municipal consultants agree that the GAME Houses have contributed to creating a more diverse range of sporting opportunities within the municipalities, and that the facilities function effectively as a platform for promoting these opportunities to children and young people. Part of their legitimacy, therefore, also lies in the GAME Houses' central role in supporting a diverse and pluralistic sports culture within the municipalities.

## Discussion

This study shows that making space for street sport, such as with the GAME House concept, can generate public value for local street sport communities. Yet, the findings also reveal a set of tensions that are important to address when interpreting the broader societal implications of such facilities. Three themes stand out as particularly significant: the institutionalization of street sport, the gap between formal governance structures and actual participatory practices, and the relational and context-dependent nature of public value creation.

### The institutionalization of street sport

A central theme concerns what happens when street sport becomes institutionalized. Street sport is historically and culturally tied to resistance, informality, and spontaneity (30) and its growing recognition as a tool for social change is closely linked to its alternative, self-organized character—precisely because it unfolds outside the structures of traditional sport (3, 29). This raises the question of whether this transformative potential risk being diluted if street sport increasingly becomes something one “attends” in designated facilities, mirroring the logic of mainstream organized sport.

Yet the findings complicate any simple narrative of taming. It is striking that practitioners themselves strongly desire stable, indoor facilities and place a high value on being recognized as legitimate actors within both the local community and municipal sport policy. Our analysis shows that culturally attuned, purpose-built facilities matter greatly to these groups, particularly in enabling year-round continuity and in attracting new participants. Previous research has similarly shown that practitioners are often willing to compromise aspects of their practice simply to gain access to indoor space (25, 31). However, the GAME Houses appear to offer a model that not only provides shelter but actively aligns with the cultural logics of street sport, combining architectural atmosphere with flexible collaboration agreements that respect local identities.

A limitation of our study is that it does not include local groups who may have actively chosen not to engage with the GAME Houses. Groups with a more resistant or anti-institutional orientation might not have welcomed the spatial fixation and formalization that the GAME Houses represent. Their perspectives could offer a more critical view of the cultural transformation these facilities introduce. Nevertheless, the findings make clear that it is overly simplistic to interpret the institutionalization of street sport as either a threat or a benefit. The facilities do not merely “tame” street sport; they also create new opportunities for community-building and cultural recognition. At the same time, this transformation requires careful attention to avoid eroding the autonomy and identity that make street sport meaningful to many participants.

### Governance—formal participation versus lived collaboration

A second key theme concerns the discrepancy between the formal governance model envisioned in the project documents and the realized practices. Although the user council model was developed by GAME to suit the local groups and ensure their democratic influence and co-creation, it reflects a conventional democratic governance principle: a formalized, representative body designed to ensure user influence. The findings of this study show that this model did not take root in any of the three facilities. Instead, collaboration unfolded through informal, relational and highly localized practices. As one manager explained, “you simply have to figure out what works locally,” underscoring the need for adaptive governance rather than fixed organizational templates.

This outcome aligns with research showing that informal and lifestyle sport communities often struggle to operate within formal institutional structures. King and Church (12) demonstrate that self-organized groups frequently find it difficult to engage with governance models designed around stability, hierarchy and predictability—features that do not match the flexible and spontaneous nature of their activities. Similarly, Jeanes et al. (11) argue that many sport facilities are “too structured and exclusive” for informal sport cultures, which require low-threshold, fluid and culturally resonant environments. The present findings extend this critique to the organizational domain: not only must physical spaces be flexible, but governance structures must also reflect the organizational logics of the communities they aim to include.

Forsberg and Iversen (8) show that managers of traditional facilities often by default prioritize established sport associations, even when they aim to create value for all citizens. The initial ambition to establish a formalized user council in the GAME houses illustrates how deeply embedded such institutional logics are in public sport provision. Our research on the GAME Houses shows, however, that their success did not depend on implementing formal participatory structures, but on the ability of local managers to cultivate trust-based, adaptive and culturally attuned forms of collaboration that resonate with street sport communities.

Yet these findings also invite reflection on the extent to which the GAME House model departs from the prioritization logic identified by Forsberg and Iversen (8). While the GAME Houses engage in collaborations with sport associations, they simultaneously broaden the range of actors recognized as legitimate partners in the development of activities and the everyday life of the facilities. The key shift therefore lies not in abandoning the collaborative partnerships associated with this prioritization logic, but in redefining who can be recognized as a legitimate partner within publicly funded sport provision.

### Public value as relational, negotiated, and context-dependent

Finally, the findings underscore that public value in the context of street sport is not a stable or easily measurable outcome, but an emergent property of relationships between users, municipal actors, and the GAME organization. The value created is both material—access to high-quality, purpose-built facilities—and normative, as users experience recognition, legitimacy, and a sense of belonging. For example, the graffiti community emphasized that legal walls allow them to practice without being “criminal just for painting graffiti,” illustrating how the facilities contribute to broader deontological notions of justice and inclusion.

However, this value is contingent on a delicate balance of operational capacity, legitimacy, and local engagement. While the daily managers in each GAME House play a decisive role in adapting organizational practices to local needs—ensuring that diverse groups not only feel welcome but actively choose to use the facilities—this local responsiveness is only one part of the equation. A significant source of legitimacy and political support stems from GAME's long-standing national expertise in street sport and youth engagement. Municipal actors repeatedly emphasized that GAME brought knowledge, credibility, and professional experience that the municipalities themselves did not possess. This expertise has shaped municipal understandings of street sport more broadly and has been central to securing long-term political and financial support for the facilities.

This raises an important point for international transferability: not all countries have a comparable central actor with the organizational capacity, reputation, and accumulated know-how that GAME represents in Denmark. The public value generated by the GAME Houses is therefore not solely a product of local co-creation, but also of a national organizational infrastructure that lends authority, stability, and strategic direction. In contexts where such an actor does not exist, similar initiatives may struggle to achieve the same level of legitimacy or to navigate the complex relationships between informal communities and public institutions. Public value in this field is thus deeply relational—not only between local actors, but also between local practice and national organizational capacity.

## Limitations

Finally, it is important to acknowledge the limitations of the study. The evaluation of the three GAME facilities was conducted between 2018 and 2020, and the data therefore represent a snapshot of value creation during this period. Since then, new users and user groups have emerged, each contributing to the ongoing development of the local facilities. Nevertheless, the study offers timely and substantive insights into the institutionalization and governance of street sport, as well as into public value understood as relational, negotiated, and context-dependent.

It is also important to note that the article focuses on investigating how the GAME House concept fosters public value by engaging local street sport communities that are typically excluded from conventional sports infrastructures. Consequently, the study does not capture the full scope of public value generated by the facilities. However, the analysis does incorporate a municipal perspective, represented by consultants and directors from the Culture and Leisure Departments in the three municipalities. To examine the full public value produced by the facilities, future research would benefit from including the perspectives of individual users who utilize the houses for self-organized training. Because the study focuses on user groups that had chosen to establish formal collaborations with the GAME Houses, the findings are also likely to reflect groups that view the facilities relatively positively. Consequently, the levels of legitimacy and support identified in the analysis may be higher than would be the case if non-participating or more anti-institutional groups had been included.

## Conclusion

In this analysis, we have examined how the GAME House concept creates public value by providing purpose-built facilities for street sports, and how this value depends on both institutional support and the facility's capacity for ongoing operational adaptation. Drawing on Moore's strategic triangle, the analysis shows that GAME Houses generate public value by meeting the practical and cultural needs of street sport communities otherwise marginalized within traditional sports infrastructures, by strengthening local communities, and by publicly recognizing street sports and street cultures as legitimate and valuable forms of participation. Importantly, this value is not solely embedded in either the physical facilities or the formal governance structures, but emerges from the interaction among spatial design, cultural atmosphere, and ongoing organizational adaptation to local contexts. The findings demonstrate that flexibility—rather than strict adherence to predefined models of user involvement—and recognition of local street sport communities as experts in their own practices have been crucial for sustaining legitimacy and support among diverse user groups. In this respect, our findings align with Jeanes et al. (11), who emphasize the importance of collaborative approaches to the development and management of facilities for informal sport.

The analysis also suggests that public value creation depends not only on local adaptation and user engagement, but on the existence of a broader organizational infrastructure capable of providing legitimacy, expertise and long-term political support. This finding raises important questions about the transferability of the GAME House model to contexts where no comparable national actor exists.

The findings also show that the public value generated by the GAME Houses operates across multiple ethical perspectives and at several societal levels. From a utilitarian level, the facilities provide functional, safe, and relevant spaces for practice. From a deontological level, they create recognition, inclusion, and the right to participate in urban cultural communities. Beyond these dimensions, the GAME Houses generate public value at different scales; they support specific groups by offering dedicated spaces and cultural legitimacy, while also contributing to a broader public value by exposing children and young people to a more diverse and culturally varied landscape of physical activity within the municipality. In this sense, the GAME Houses generate both material welfare and normative value, while simultaneously expanding the cultural horizons of local sport ecosystems.

Finally, the analysis points to important tensions and limitations inherent in the GAME House concept. The institutionalization of street sports through publicly governed, purpose-built facilities risks diluting some of their historically self-organized and DIY-oriented characteristics. For certain practices, the transition from informal, self-produced spaces to publicly governed facilities may constitute both a gain in accessibility and legitimacy and a loss of autonomy and subcultural distinctiveness. These tensions underline that public value creation in facilities for street sports is inherently fragile and contingent, requiring continuous negotiation between inclusion, institutional support, and the preservation of the alternative qualities that make street sports distinctive in the first place.

## Data Availability

The datasets presented in this article are not readily available due to the sensitive and potentially identifiable nature of the interview data. The original interview data have been deleted in accordance with applicable data protection requirements. Further inquiries can be directed to the corresponding author.

## Footnotes

^1^To read about GAME: https://game.ngo

^2^To read about GAME House Copenhagen: https://game.ngo/where-we-work/denmark/game-house-copenhagen

^3^Detailed, technical documents—including drawings, blueprints, and specifications—that outline the precise instructions, materials, management and structural details required to build a project.

^4^The comprehensive evaluation conducted between 2018 and 2020 aimed to investigate the social effects on target groups, the interaction between facilities, architecture, and activities within the local community, as well as the user involvement process and the development of local ownership. The full evaluation report can be found here (in Danish): https://www.sdu.dk/da/forskning/cisc/forskningsomraader/forskningsprojekter/projekter/2017_game_streetmekka

^5^Since 2018, the skate area in Esbjerg has undergone redevelopment in close collaboration with the local skate community, ensuring alignment with local needs.

^6^According to the national database for sports facilities, there are 77 parkour facilities and 148 skateboarding facilities owned or managed by municipalities across Denmark's 98 municipalities. Database: https://www.idan.dk/projekter/facilitetsdatabasen/
